# Medical Association of Ga.

**Published:** 1886-03-20

**Authors:** 


MEDICAL ASSOCIATION OF GEORGIA.—This Association will meet in Augusta on the third Wednesday in April next. A large attendance and a pleasant time is anticipated. Augusta is noted for the generous hospitality of her citizens, and the medical profession in that city contain amongst its members some of the first men in the country, and on all occasions in which they have been called upon to entertain the Association they have done it in a wholesoul and generous way.

